# How does law and policy support providers of NHS healthcare in England to respond to harm experienced by patients during the course of their treatment and care: A scoping review

**DOI:** 10.1371/journal.pone.0347997

**Published:** 2026-04-30

**Authors:** Naomi Assame, Susan Greenhalgh, John Tingle, Gillian Yeowell

**Affiliations:** 1 Manchester Metropolitan University, Manchester, United Kingdom; 2 Manchester Metropolitan University, Manchester, United Kingdom; 3 University of Birmingham, Manchester, United Kingdom; 4 Manchester Metropolitan University, Manchester, United Kingdom; Universidade de Lisboa Instituto Superior de Ciencias Sociais e Politicas, PORTUGAL

## Abstract

**Introduction:**

Many countries have implemented laws and policies to help healthcare providers address patient harm. However, there is limited research on their effectiveness, especially in the context of England#39;s National Health Service (NHS). This scoping review asks: How does law and policy support NHS healthcare providers in England respond to harm?.

**Methods and analysis:**

A scoping review methodology was applied to map and summarise the literature to date. MEDLINE, CINAHL, and Westlaw were searched to identify potentially relevant literature that had been published between 2000 and 2025. Literature was included if it addressed care delivery in England#39;s NHS and healthcare providers’ responses to patient harm. Two reviewers independently screened the literature identified from the search against the eligibility criteria. A grey literature search of the citations within the included records was then conducted to identify relevant law and policy that supports providers of NHS healthcare in England to respond to patient harm. A total of 121 records were reviewed: 63 peer-reviewed and 58 pieces of grey literature. Data were charted using a standardised data extraction form. Themes were developed through a combination of thematic and content analysis, then presented as a narrative synthesis.

**Conclusion:**

The first of its kind, this scoping review identified English laws and policies that support NHS healthcare providers in England to respond to patient harm. It revealed the incongruent yet interdependent relationship that exists between England’s legal systems, healthcare policies and the promoted NHS values. The scoping review highlighted how organisational culture can overshadow law and policy and be the defining factor in how providers of NHS healthcare in England respond to patient harm. The impact of law and policy in curating organisational culture and response to patient harm is an area requiring further research.

**Ethics:**

Ethics approval was not required for this review.

## Introduction

Globally, around one in 20 patients are exposed to harm in medical care due to failures in the healthcare system or a combination of errors made by individuals, system failures and patient characteristics [1]. In addition to the emotional, physical and psychological costs to the patient and their family, there are significant societal and economic costs due to reasons such as additional morbidity requiring additional care or hospital admissions(s), and reductions in economic productivity due to absence from work [2].

Countries around the world have adopted legal frameworks and policies to support healthcare providers to respond to patient harm. Gil-Hernandez [3] conducted an international, cross-sectional study examined these mechanisms across 27 countries and revealed significant variance. Less than half of the participating countries had a specific agency or institution responsible for leading a patient safety strategy. Over two-thirds of participating countries reported the absence of a specific policy or legislation regulating the systematic duty to openly disclose adverse events to patients. Three-quarters of the countries surveyed stated that no compensation scheme existed to support those affected by patient harm. The United Kingdom (UK) was not a country included in this study. Gil-Hernandez [3] concluded that future studies should delve deeper into how these mechanisms support providers to respond to patient harm, not only with a focus on compensation costs, but also in terms of improving patient safety.

In the United Kingdom, the government finances the National Health Service (NHS) through taxation, ensuring that all residents have access to comprehensive medical services free of charge at the point of use. In 2023, the UK was ranked 21^st^ out of 38 in the Organisation for Economic Co-operation and Development (OECD) countries for patient safety [4]. The government in the UK has devolved its powers to its four constituent nations, i.e., England, Wales, Scotland and Northern Ireland. Therefore, each nation has its own policies, governance frameworks, legal frameworks and mechanisms for responding to patient harm, including the handling claims for clinical negligence. As the most patient safety incidents occur in England, this review will focus on English law and policy only.

It is estimated that improving how the NHS in England responds to harm could save 928 lives per year and release £98.5 million more for care annually [5]. Reducing the risk of patient harm associated with the delivery of NHS healthcare is a policy priority that is linked to the United Kingdom (UK) government’s desire to drive down costs associated with claims for clinical negligence brought against the NHS in England. In 2023/24, £2.8 billion was paid out for clinical negligence claims [6].

In 2018, NHS Resolution the organisation that indemnifies the NHS in England, published research that identified that those pursuing a claim for clinical negligence will do so out of frustration because of the absence of a transparent investigation and apology from the healthcare provider of concern [7]. This is despite the presence of law and policy articulating the legal, ethical, and moral requirement to be open, honest, and transparent when harm has occurred including providing an apology.

Law and policy are key to supporting English providers of NHS healthcare to improve their response to patient harm but are not necessarily well understood, therefore can be challenging to apply to practice. Greater understanding will improve response to patient harm and ensure those affected receive the justice they deserve as well as protect the public purse. However, there is a paucity of research exploring how English law and policy underpins response to harm thus limiting understanding. To address this gap in knowledge, a scoping review was undertaken. The aim was to investigate how law and policy support providers of NHS healthcare in England to respond to harm experienced by patients during the course of their treatment and care.

## Methods and analysis

Scoping review methodology aims to map and summarise the literature to date [8]. To address the above aim, the Arskey and O’Malley six-stage framework for conducting scoping reviews [8] was adopted as per our protocol [9]. To enhance the framework, the recommendations of Levac [10] and the Joanna Briggs Institute (JBI) [11] were incorporated as per our protocol [9]. The review was registered via the Open Science Framework (https://doi.org/10.17605/OSF.IO/8HF9C) on the 19^th^ of September 2022.

### Stage 1: Identifying the research question

The research question for this scoping review is: **How does law and policy support providers of NHS healthcare in England to respond to harm experienced by patients during the course of their treatment and care.**

### Stage 2: Identifying relevant literature

#### Search strategy.

Database search: Relevant published literature was identified by searching the peer-reviewed databases MEDLINE, Cumulative Index to Nursing and Allied Health Literature (CINAHL) and Westlaw. The search strategy included a combination of the following keywords and where appropriate corresponding MeSH terms, using Boolean operators such as And and Or: National Health Service, apology, compensation (see Table 1).

**Table 1:** Complete MEDLINE and CINAHL search strategy.

	Keywords / search terms
#1	NHS OR “National Health Service” OR healthcare OR “health care”
#2	Apology OR “saying sorry” OR “duty of candour” OR complaint OR investigation OR compensation OR sue OR claim OR litigation OR “clinical negligence claim” OR “alternative dispute resolution” OR mediation OR inquiry OR inquest Or Fault OR “no fault”
#3	Incident OR “serious incident” OR “medical error” OR “never event” OR “avoidable injury” OR “preventable injury” OR “avoidable harm” OR “preventable harm” OR “patient safety incident”
#4	#1 AND #2 AND #3

The search was conducted on the 18^th^ of December 2023 and re-ran on 21 March 2025 to identify any additional records. The date range applied to the search strategy was for academic studies published between 2000 and 2025.

### Stage 3: Study selection

Records retrieved from the search were screened and included if they met the following criteria:

- Concerned the delivery of care by the NHS in England
- Considered how healthcare providers respond to patient harm
- Considered approaches to investigating patient harm
- Considered approaches to the reduction of patient harm
- Refers to statute, policy or case law

Records were excluded if they met any of the following criteria:

- Concerned the conduct of a health professional, dishonesty, fitness to practice
- Concerned the breach of data protection laws
- Concerned the measurement or incidence of patient harm
- Concerned the classification of patient harm
- Established the types of injury that arise from patient harm
- Concerned the assessment, diagnosis, treatment or prevention of a clinical pathology
- Concerned measuring the prevalence of clinical pathology
- Concerned harm to health care staff

The JBI guidance on scoping reviews [11] recommends that two or more reviewers screen articles independently. However, where this is not possible, for example, due to resource constraints, others have recommended that one reviewer conduct an independent review, with a second reviewer verifying a proportion of papers with a goal of 90% agreement [12]. Guided by the above inclusion and exclusion criteria, the title and abstract were screened independently by one reviewer (NA). If uncertainty existed around eligibility, the full text of potentially relevant records was reviewed. A second reviewer (GY) completed the same process for 20% of the records extracted. A third reviewer (SG) was available to resolve any disagreements regarding the inclusion of records. There was concordance of 100% between the two reviewers. A grey literature search of the citations within the included records was then conducted to identify relevant law and policy that supports providers of NHS healthcare in England to respond to patient harm.

### Stage 4: Charting the data

A charting from was developed using parameters based on those described within the JBI Manual for Evidence Synthesis [11]. Development of the charting form was an iterative process as the review team continued to extract the data, became more familiar with the results extracted and updated the form accordingly. To ensure all relevant results were extracted, the charting form was piloted by different members of the review team and amended accordingly. The charting form included the inclusion criteria and explained the rationale for excluded data (S1 F*ile)*.

### Stage 5: Collating, summarising and reporting the results

To identify, analyse and report the themes within the data extracted from the included literature, the principles of thematic analysis outlined by Braun and Clarke [13] were drawn upon to identify initial codes. This required a five-phase process consisting of familiarisation with the literature (phase one), generating initial codes relevant to the research question, (phase two), identifying contextual themes (phase three) measuring the frequency of initial codes (phase four) and using the initial codes that occurred the most frequently and their contextual theme to iteratively and inductively derive the central themes of the data set (phase 5). Given the high volume of records selected for inclusion in the scoping review, the quantitative element of content analysis was incorporated into the methodology. The quantitative element of content analysis counts the frequency of codes to identify recurring patterns. It is an approach which is recognised as being helpful to identify patterns and emerging themes within large data sets [14,15]. Whilst coding was performed by a single reviewer, rigor of the evidence synthesis was ensured through regular team meetings to discuss the review process including the data extraction, analysis, and presentation [1x]A narrative synthesis was then performed to synthesise the findings of the included records.

Ethics approval was not required for this scoping review. All records were obtained from publicly available documents, and no primary data was generated.

## Results

### Descriptive analysis

**Fig 1:**
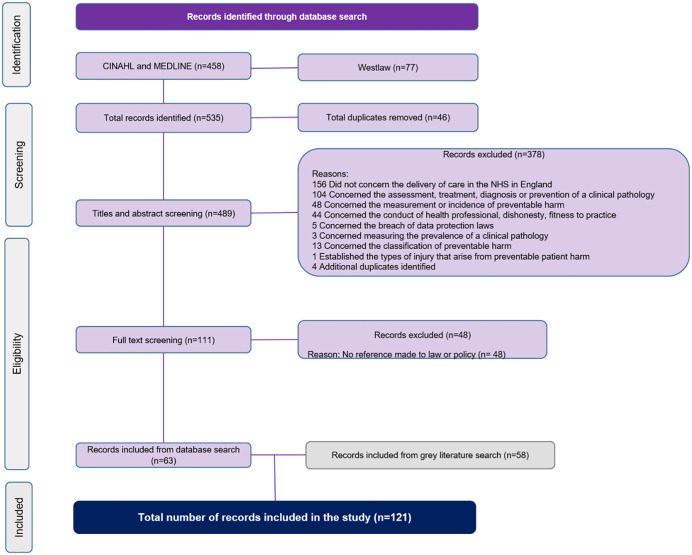
Scoping review flow chart.

The flow diagram (Fig 1) shows the results of the search, and the number of records found. A total of 535 records were identified, 472 were excluded leaving 63 records being included in the scoping review. The bibliographic citations of the included literature were searched to identify further relevant literature that referenced law and policy that supports providers of NHS healthcare in England to respond to patient harm. This search yielded a further 58 pieces of grey literature. The total items of literature included in the scoping review were 121.

**Fig 2:**
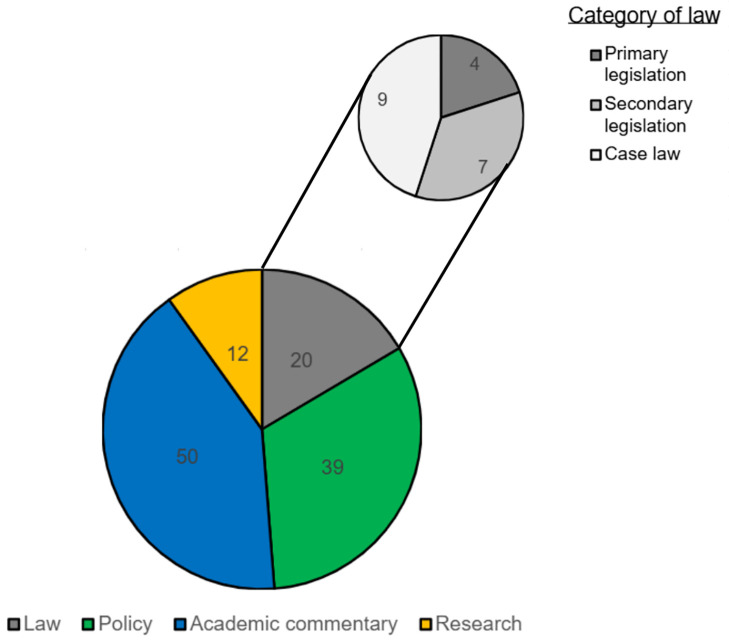
Volume of records by classification of publication.

Charting of the records included in the scoping review captured the year of publication, the classification of the record extracted and key findings (S1 F*ile)*. Records were classified as either law, policy, academic commentary, or research. In the context of this scoping review, ‘law’ is categorised into case law (i.e., a set of rulings from court judgements that set precedents for how the law has been interpreted and applied in certain cases), primary legislation (i.e., main laws passed by the legislative bodies) and secondary legislation (i.e., delegated legislation made by a person or body under authority contained in primary legislation). Fig 2 shows the volume of records falling under each classification and includes a further breakdown by category of law.

**Fig 3:**
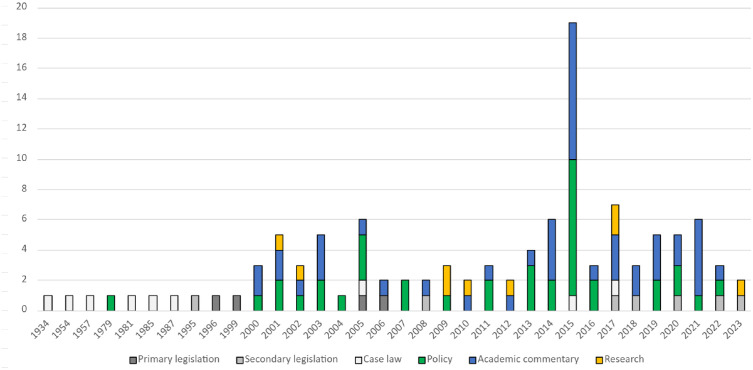
Distribution of records by year of publication and classification.

Fig 3 shows a graphical representation of the distribution of records by year of publication and classification.

### Narrative synthesis

**Fig 4:**
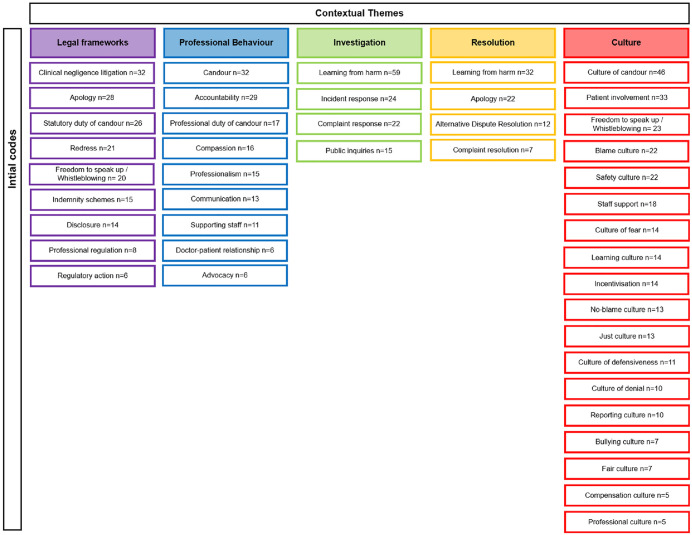
Summary of initial codes and contextual themes.

Following familiarisation with the records, 36 initial codes relevant to the research question were identified from the 121 publications included in the scoping review. The records referenced under each initial code were tabulated and can be found in S2 File. The codes were categorised under five contextual themes. Of the five contextual themes generated, culture held the greatest number of initial codes (n = 18), followed by legal frameworks and professional behaviour (n = 9), and Investigation and Resolution (n = 4) (Fig 4).

The narrative findings for each contextual theme are presented below

#### Legal frameworks.

This contextual theme describes legal frameworks that shape how providers of NHS care in England respond to patient harm. Primary legislation requires all employers in England, including the NHS, to provide a safe place of work and investigate harm when identified. Failure to comply can result in regulatory action being taken against organisations [16]. English law illustrates its support for addressing patient harm by requiring transparency and honesty with patients following a notifiable patient safety incident, which includes offering an apology [17]. Legal frameworks protecting healthcare professionals who choose to speak up about patient safety concerns were identified within the records extracted [18]. Records extracted also revealed the legal frameworks such as statutes and case law that underpin how compensation is decided for harm arising from harm caused by the NHS in England [19,20,21,22]. Whilst English case law is of influence internationally, it can only be transferred to those countries which use common law systems such as Ireland, Australia and New Zealand.

#### Professional behaviour.

This contextual theme describes the behaviours English laws and policies expect health care professionals to exhibit when responding to patient harm. The standards professional regulators place upon their registrants when patient harm occurs were identified from the records extracted. This includes the expectation that registrants will support investigation processes and be open, honest, and apologise when patient harm occurs [23,24,25]. The strong oversight regulatory councils provide to its registrants England is not consistently replicated across the world. The records extracted identified English healthcare policy defining accountability as the requirement of healthcare leaders to ensure systems oversight is in place. This includes ensuring that resources are used appropriately to understand incidents, implement changes, share learning across the system, and ensure an honest response to patients and families affected by patient harm [5,26]. Compassionate engagement with patients and families who have been affected by harm, including healthcare staff, is a central tenet to English healthcare policy [5]. Compassion is also a behaviour that is considered by several academic commentators to be an important value for healthcare professionals to exhibit towards patients, families, and staff [27–29]. The importance of effective communication with those affected by patient harm was described by academic commentators across the records extracted [30–32]. Analysis of the records identified the existence of English law and policy that supports providers of NHS healthcare to communicate effectively when responding to patient harm [5,17,33].

#### Investigation.

This contextual theme describes how English law and policy supports providers of NHS healthcare in England to investigate complaints and incidents in a way that learning is identified and implemented. Several laws that enable this were identified from the record extraction [16,17,34–37]. Healthcare policy, including a national strategy for patient safety, was also identified as supporting providers of NHS care in England to investigate patient harm effectively by using patient safety science methodologies and involving patients, families and staff [5,33,38]. The importance of using the investigation process to learn from harm was referenced across the records extracted [28,30,31,39,40].

#### Resolution.

This contextual theme describes the different processes followed and actions taken to ensure those affected by patient harm receive the resolution they are seeking. Across the extracted records, learning from harm was referenced as a resolution wanted by those affected by patient harm [41–44]. The presence of healthcare policies that support providers of NHS care in England to learn from patient harm were identified [5,33,39]. Receiving an apology for the harm experienced was also referenced as a key resolution for those affected by patient harm [27,32,42,44–46]. Alternative Dispute Resolution (ADR) was identified in the records as a mechanism for resolving claims for compensation outside of the court system and is an approach supported by English law [19]. ADR was described within the records as being an effective way of enabling the clinical negligence process to focus on what matters to a particular patient, reducing the burden placed upon providers of healthcare, as well as reducing legal costs [46–48].

#### Culture.

This final contextual theme describes the differing values, beliefs, and behaviours displayed by healthcare professionals working within the NHS in England. A range of cultures were described across the records extracted for this scoping review. English policies were noted to aspire to ensuring ‘just’ and ‘learning’ cultures exist within providers of NHS healthcare in England [5,38]. Whilst the Statutory Duty of Candour [17] aligns within this ambition, academic commentators considered the legal system in England to focus on the acts of individuals rather than systems thereby driving cultures of blame within the NHS in England [49]. Academic commentators described the importance of involving patients in the response to harm as this provides evidence of an organisation’s commitment to openness, accountability, supporting individual need, and rebuilding trust [50]. This approach is endorsed within English policy aimed at supporting providers of NHS care in England to respond to patient harm effectively [5,38].

**Fig 5:**
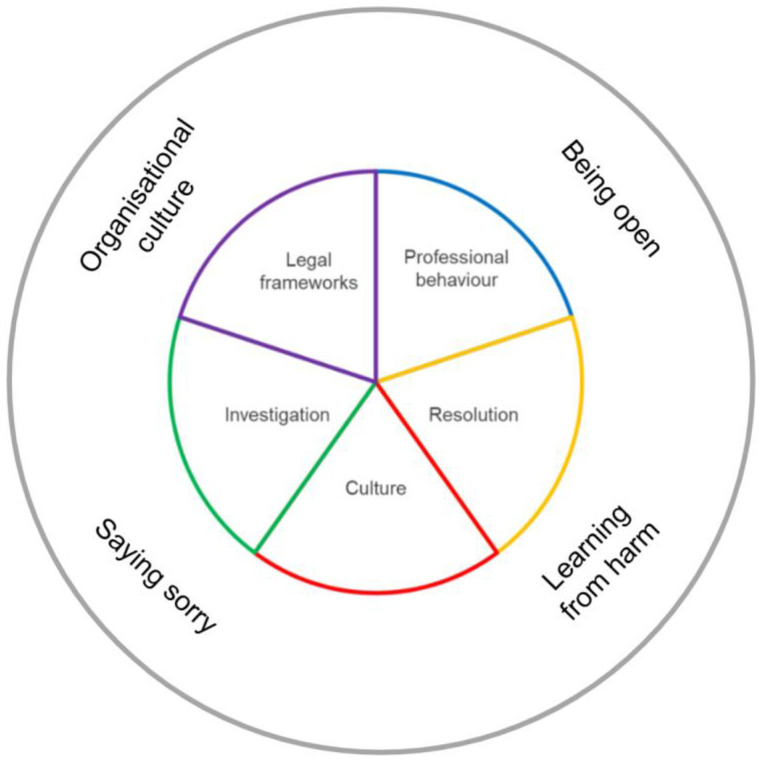
Main concepts related to contextual themes.

To complete the synthesis, the contextual themes were considered by the review team and refined to derive the main concepts that encapsulated the essence of the extracted data. Four main concepts were identified: ‘Organisational culture’, ‘Saying sorry’, ‘Being open’ and ‘Learning from harm’ (Fig 5). These four conceptual themes are of relevance to each of the five contextual themes which are not mutually exclusive.

## Discussion

This scoping review investigated how law and policy supports providers of NHS healthcare in England to respond to harm experienced by patients during the course of their treatment and care and represents the first review of its kind. Four main themes were identified: ‘Organisational culture’, ‘Being open’, ‘Saying Sorry’, and ‘Learning from harm’.

### Organisational culture

Culture held the greatest number of initial codes across the five contextual themes indicating the significant influence organisational culture has on how harm is responded to.

The records extracted identified the existence of laws that support providers of NHS healthcare in England to respond to patient harm [16–19]. However, policy documents and academic commentary identified through the data extraction suggested that cultures of blame, fear and denial could be attributed to how healthcare providers perceive and experience the application of law relating to response to harm.

*Organisation with a memory* [39] was the first policy document to discuss the importance of developing organisational cultures that are open and allow harm to be admitted, reported, and discussed. The Bristol Inquiry [51] identified fear of blame, whether in the press or through litigation, as being a powerful inhibiting factor to creating an open culture within the NHS. Case [52] attributed the adversarial nature of litigation and its propensity to individualise blame to the genesis of blame cultures that result in defensiveness.

The Mid-Staffordshire Inquiry [53] reported an organisational culture where staff were frightened for their own jobs and if they did speak up, senior staff were hostile towards them, pressurising them to drop their concerns or leave the organisation [27]. Subsequent public inquiries have made similar observations [54–56]. Despite the Employment Rights Act [18] protecting staff who raise concerns from suffering detriment, it appears that organisational culture can be so powerful, it can overshadow the legal powers that are in place to protect those who raise concerns about patient safety. Freedom to speak up and whistleblowing were identified as important elements of the legal frameworks contextual them within this scoping review.

The records extracted for this scoping review have revealed a multitude of policy documents aimed at supporting providers of NHS healthcare in England to embed cultures that support an effective response to patient harm [5,40,54,57–59]. Despite the presence of these policies, embedding such cultures continues to be a pervasive challenge across the NHS in England. Current law and policy alone do not appear to be able to address this issue.

Solutions proffered within the records extracted for this scoping review include ensuring that the processes in place to support response to patient harm are truly non-punitive [60], ensuring a systems approach is consistently taken to understand the factors contributing to patient harm [61] and that staff involved in any aspect of patient harm and the resulting response are adequately supported [62].

### Being open

Candour was an initial code that frequently occurred in the records extracted for this scoping review. The Professional Standards Authority [62] define candour as “being open and transparent when something has gone wrong”. As discussed, the culture of an organisation impacts the ability of those they employ to be open and in part, this is influenced by relevant law and policy.

The English courts first considered the topic of candour within the context of healthcare and a medical professional#39;s duty to disclose medical error in the case of Gerber *v* Pines [20]. In this case, the judge concluded that the doctor did have a duty of care to immediately disclose the error to the patient and failing to do so was a breach of duty. However, the judge also expressed their opinion that there may be circumstances where it may *not* be considered a breach of duty to fail to disclose a medical error to the patient. Specific examples were not provided by the Judge but the decision in Lee *v* South Thames Regional Health Authority [21] indicated that if such a circumstance could be where disclosure it may place those responsible at risk of litigation. This decision was possibly indicative of the court’s deference to the medical profession and medical protectionism that existed at that time. However, when summing up the decision of the Court of Appeal, Sir John Donaldson Master of the Rolls did suggest there may also be circumstances where withholding certain information may protect the patient from further harm.

*Making amends* [58] suggested statutory provisions be introduced to encourage openness including placing a professional duty on clinicians and healthcare managers to inform patients of actions resulting in harm and protecting those reporting harm from disciplinary action. Whilst policy documents such as ‘*Being open’* [33] provided guiding principles for healthcare organisations to follow to encourage openness, it was not until ten years after *Making amends* [58] was published that statutory and professional duties were introduced. In 2014, the statutory duty of candour [17] was brought into law placing a legal requirement upon every health and social care provider that the Care Quality Commission (CQC) regulates to be open and honest with those receiving care.

The General Medical Council (GMC) was the first regulatory body to place a professional duty upon its registrants to uphold the principles of candour in its *Good medical practice* guidance [23]. The Nursing and Midwifery Council (NMC) followed suit in 2015 [63] and The Health and Care Professions Council (HCPC) in 2016 [24]. Breaches of professional standards can amount to misconduct meaning that any health professional found to not be upholding the principles of candour could be subjected to the fitness to practice process. However, an observation of Glasper [27] is that because the statutory duty of candour does not afford protection from disciplinary action, healthcare professionals may be reluctant to be open and as they may fear retribution from their employer and professional regulator.

A further consideration is that the law of negligence requires proof that there has been a breach in professional standard. This may surmount to misconduct and result in the healthcare professional being subjected to fitness to practise processes. Such consequences are quite possibly a deterrent to healthcare professionals being open and honest. Whilst the findings of this scoping review have identified the presence of law and policy supporting providers of NHS healthcare in England to be open about patient harm, a juxtaposition exists across legal frameworks resulting in a potential candour conundrum prompted by healthcare professionals fearing disciplinary or civil action as a result of being open.

### Saying sorry

There is a general societal expectation that an apology will be given to those who are harmed. Providing an apology in the wake of patient harm was identified from this scoping review within the contextual themes of legal frameworks and resolution. The records extracted identified the existence of law that requires providers of NHS healthcare in England to apologise for patient harm.

The Compensation Act [19] makes provision about arrangements for redress in relation to any liabilities that may arise through the delivery of the health service in England or Wales. In addition to the provision of financial compensation and providing an explanation, the Act requires an apology to be given. The statutory duty of candour [17] states that where a notifiable patient safety incident has occurred, an apology should be provided.

Analysis of the records extracted for this scoping review identified the presence of policy documents that support providers of NHS healthcare in England to apologise for patient harm. *Being open* [33] clearly states that saying sorry is not an admission of liability, is the right thing to do and is fully supported by the organisation responsible for indemnifying the NHS in England. This notion has been re-enforced more recently in the NHS Patient Safety Strategy [5].

Despite the presence of law requiring providers of healthcare to apologise, the records extracted for this scoping review suggested that individuals may be discouraged from apologising in case they inadvertently make an admission of liability or potentially facing professional regulatory action because of admitting a breach in professional standards [41,45,64,65].

### Learning from harm

Learning from patient harm was identified from this scoping review within the contextual themes of resolution and investigation. Learning from patient harm was also linked to the ‘Culture of learning’ within the contextual theme of ‘culture’.

The record extraction for this scoping review revealed the plethora of policy supporting learning from harm [5,38,22,66] yet identified the absence of a comparable legal framework. Analysis of the records revealed the disconnect that exists between legal frameworks and policy approaches to establishing the circumstances that led to patient harm. Relevant policy promotes systems thinking whereas the law of negligence focusses on the actions of individuals.

Models of healthcare delivery within the modern NHS in England are far more complex than those in place when the courts heard Bolam v Friern Hospital Management Committee [22]. The law of negligence looks to establish whether harm was caused by the actions of the healthcare professional. It does not consider the complex healthcare system within which the healthcare professional is operating, and the competing demands they are facing. Taking a systems approach to learning from harm and improving patient safety is a key philosophy underpinning England’s Patient Safety Strategy [5]. The findings of this scoping review suggest that legal frameworks supporting providers of NHS healthcare in England to respond to harm have not evolved to align with current thinking around patient safety. It is possible that this is having an inhibitory effect on enabling learning from harm.

### Strengths and limitations

There are strengths and limitations associated with this scoping review. Utilising the scoping review methodology has enabled the systematic search of the MEDLINE, CINAHL and Westlaw databases to systematically analyse existing records and identify relevant grey literature to answer the research question. Synthesis of the records has enabled the identification of gaps in current knowledge and understanding and provided direction for future research.

A potential limitation is that a single reviewer screened 80% of the records. Nevertheless, the 20% of records that were independently screened by a second reviewer demonstrated 100% concordance, which strengthens the reliability and credibility of the analysis. Whilst there is no formal assessment of the methodological quality of the literature included in a scoping review [66], a benefit of the scoping review methodology is that the review question can be broader than those developed for systematic reviews

Law and policy relevant to how providers of NHS healthcare in England respond to patient harm continuously evolve. To an extent, frontline awareness of associated changes, and their practical application, is dependent upon the publication of relevant academic commentary. The impact of this limitation is that recently published law and policy relevant to the research question may not have been identified through the data extraction.

## Conclusion

This scoping review is the first to investigate how law and policy supports providers of NHS healthcare in England to respond to harm experienced by patients during their treatment and care. This scoping review has identified the presence of law and policy aimed at supporting providers of NHS healthcare in England to respond to patient harm. Previous research examining the mechanisms in place to support healthcare providers around the world to respond to patient harm did not include England [3]. This scoping review has demonstrated that England has established a comprehensive national strategy for patient safety, along with specific legislation and policies governing the open disclosure of patient safety incidents. Additionally, a compensation system is in place to support individuals impacted by patient harm. Other countries may wish to consider how they could mirror how English law and policy supports providers of healthcare to respond to patient harm. However, caution should be applied as this scoping review has revealed the incongruent yet interdependent relationship that exists between England’s legal systems, healthcare policy and the promoted NHS values. Despite their presence, embedding such cultures continues to be a pervasive challenge across the NHS in England. Current law and policy alone do not appear to be able to address this issue.

Analysis of records extracted for this scoping review has highlighted how organisational culture can overshadow law and policy and be the defining factor in how providers of NHS healthcare in England respond to patient harm. Whilst the intention of law and policy may be to improve response to patient harm, the findings of this scoping review suggest they may also be driving cultures of fear and blame. The impact of law and policy in curating organisational culture and response to patient harm is an area requiring further research. Such research is vital to inform future NHS litigation reform and optimise organisational and individual response to patient harm.

## Supporting information

S1 File S1 supplementary file. (XLSX)

S2 File S2 supplementary file. (PDF)
