# Supplementary material for: How does law and policy support providers of NHS healthcare in England to respond to harm experienced by patients during the course of their treatment and care: A scoping review
Source: PLoS One. 2026 Apr 30;21(4):e0347997. doi: 10.1371/journal.pone.0347997 (PMC13132193; doi:10.1371/journal.pone.0347997)
Supplement: S2 File — (PDF) [file pone.0347997.s002.pdf]

Supplementary file two: Tabulation of records referenced under each initial code

Table 1: Records relating to how law and policy supports providers of NHS care in England to respond to patient harm grouped into initial codes and the contextual theme of legal frameworks.

**Initial Code**	**Frequency**	**Records**
Clinical negligence Litigation	N=32	Park, S., 2005; Johnson, H. and Spencer, M., 2021; Case, P., 2020; Thomas, E. and Leigh, B., 2014; Cave, E., 2011; O'Brien, K., 2012; Tingle, J., 2021; Vernon, H., 2019; Chaffer, D., 2021; Bolam v Friern Hospital Committee., 1957; Whitehouse v Jordan,. 1981; Cruikshanks, A. and Bryden, C., 2018; Draycott, T. et.al., 2015; Henderson, N., 2001b; Carr, R., 1978; National Audit Office., 2001; The National Health Service (Clinical Negligence Scheme for Trusts) Regulations., 1996; Donaldson, L., 2003; Gregg v Scott., 2005; NHS Redress Act., 2006; NHS Resolution., 2019; NHS England and Improvement., 2019; Vincent, C. and Coulter, A., 2002; Department of Health., 2011; National Patient Safety Agency., 2005; NHS Resolution., 2022; National Patient Safety Agency., 2009; Department of Health., 2000; Robinson, A. et. al., 2025; O'Dwyer, L., 2024; Devlin, M., 2024; Kirwan, H. and McNeil, P., 2024;
Apology	N=28	Awais, Z., 2015; Vick, L., 2019; Samuels, A., 2017; Thomas, E. and Leigh, B., 2014; Glasper, A., 2021; Chaffer, D., 2021; O'Brien, K., 2012; Cruikshanks, A., 2018; Hawkes, N., 2015; National Patient Safety Authority., 2004; Department of Health., 2006; National Patient Safety Authority., 2009; Department of Health., 2009; Francis, R., 2013; Dalton, D. and Wiliams, N., 2014; NHS England., 2015; General Medical Council and Nursing and Midwifery Council., 2015; Department of Health. 2015b.; Francis, R., 2015b; Heath and Social Care Act 2008; Parliamentary and Health Services Ombudsman., 2015; Health and Care Professions Council., 2016; NHS Resolution., 2019; NHS Resolution., 2020; Getting It Right First Time and NHS Resolution., 2021; Cave, E., 2011; NHS Litigation Authority., 2001; National Audit Office., 2001;

Table 1: Records relating to how law and policy supports providers of NHS care in England to respond to patient harm grouped into initial codes and the contextual theme of legal frameworks.

Initial Code	Frequency	Records
Statutory duty of candour	N=26	Vick, L., 2019; Samuels, A., 2017; Thomas, E. and Leigh, B., 2014; Glasper, A., 2021; O'Brien, K., 2012; Edozien, L. and Clements, R., 2006; Basu, S. et.al., 2020; Cruikshanks, A., and Bryden, C; 2018; Sokol, D., 2015; Hawkes, N., 2015; Tingle, J., 2015b; Byrom, A., 2013; Donaldson, L., 2003; NHS England and NHS Improvement., 2015; Francis, R., 2015a; Health and Social Care Act 2008 (Regulated Activities) Regulations 2014: Regulation 20; NHS England and NHS Improvement., 2019; Department of Health., 2015c; General Medical Council and Nursing Midwifery Council., 2015; Francis, R., 2015b; Burn., 2003; Walker, D. and Punt, J., 2022; Awais, Z., 2015; Francis, R., 2013; Kirkup, B., 2015; Adams, M., 2024;
Redress	N=21	Cave, E., 2011; Burn, S., 2003; Edozien, L and Clements, R., 2006; Park, S., 2005; Tingle, J., 2021; National Patient Safety Agency., 2009; Gregg v Scott., 2005; Carr, R., 1978; Donaldson., L. 2003; National Patient Safety Agency., 2005; Department of Health., 2011; Parliamentary and Health Services Ombudsman., 2012; NHS Resolution., 2018; NHS England and NHS Improvement., 2015; NHS Resolution., 2019; NHS Resolution., 2022; Audit Office., 2001; National Patient Safety Agency., 2004; Department of Health., 2007; Department of Health., 2009; Devlin, M., 2024;
Whistleblowing / Freedom to speak up	N=20	Vick, L., 2019; Awais, Z., 2015; Glasper, A., 2021; Tingle, J., 2015b; Macrae, C., 2014; MacDonald, M. et. al., 2014; Byrom, A., 2013; Tasker, A., Jones, J. and Brake, S., 2023; Department of Health., 2002; Department of Health., 2007; Department of Health., 2009; Department of Health., 2015c; Francis, R., 2015b; NHS England and NHS Improvement., 2015; Parliamentary and Health Services Ombudsman., 2016; The Health and Care Professions Council., 2016; Getting It Right First Time and NHS Resolution., 2019; Royal College of Midwifery., 2022; Beatt v Croydon Health Services NHS Trust., 2017; Public Interest Disclosure Act., 1998;

Table 1: Records relating to how law and policy supports providers of NHS care in England to respond to patient harm grouped into initial codes and the contextual theme of legal frameworks.

Initial Code	Frequency	Records
Indemnity schemes	N=15	Vernon, H., 2019; Chaffer, D., 2021; Cruikshanks, A. and Bryden, C., 2018; Draycott, T. et. al., 2015; Cowan, J., 2003; Dimond, B., 2001; Henderson, N., 2001b; Donaldson., L. 2003; NHS Resolution., 2018; NHS England and NHS Improvement., 2019; Department of Health., 2011; Glasper, A., 2021; Carr, R., 1978; National Audit Office., 2001; NHS Resolution., 2022;
Disclosure	N=14	Price-Davey, A.. 2017; Gerber v Pines., 1924; The Health and Care Professions Council., 2016; Kirkup, B., 2020; Department of Health., 2000; The National Health Service (Clinical Negligence Scheme for Trusts) Regulations., 1996; Harrison, R. et. al. , 2017; Public Interest Disclosure Act., 1998; General Medical Council., 2013; Parliamentary and Health Services Ombudsman., 2016; Lee v South Thames Regional Health Authority., 1985; Adams, M., 2024; Kirwan, H. and McNeil, P., 2024; NHS England., 2022;
Professional regulation	N=8	Vosper, H., 2018; Johnson, A., 2021; Cave, E., 2011; Hawkes, N., 2015; Tingle, J., 2015b; Byrom, A., 2013; Department of Health., 2015a; O'Dwyer, L., 2024;
Regulatory action	N=6	Johnson, A., 2021; Tingle, J., 2021; Glasper, A., 2021; Basu, S. et. al., 2020; Astill, D., 2008; Health and Safety at Work etc. Act., 1974;

Table 2: Records relating to how law and policy supports providers of NHS care in England to respond to patient harm grouped into initial codes and the contextual theme of professional behaviour.

Initial Code	Frequency	Records
Candour	N=32	Edozien, L and Clements, R., 2006; Vosper, H., 2018; O'Brien, K., 2012; Tingle, J., 2015a; Tingle, J., 2015b; Kirkup, B., 2015; Pinto, V. et. al., 2012; Tingle, J., 2010; Kennedy, I., 2001; Department of Health., 2002; National Audit Office., 2005; General Medical Council., 2013; Dalton, D. and Williams, N., 2014; NHS Employers., 2014; Parliamentary and Health Services Ombudsman., 2015; Department of Health., 2015a; Nursing and Midwifery Council., 2015; General Medical Council and Nursing and Midwifery Council., 2015; Department of Health., 2015b; Department of Health., 2015c; Francis, R., 2015b; NHS England and NHS Improvement., 2015; Parliamentary and Health Services Ombudsman., 2016; NHS Resolution., 2019; NHS England and NHS Improvement., 2019; Kirkup, B., 2020; Getting It Right First Time and NHS Resolution., 2019; Royal College of Midwifery., 2022; Lee v South Thames Regional Health Authority., 1985; Department of Health., 2000; Ockenden, D., 2022; de Silva, P., 2025;
Accountability	N=29	Vosper, H., 2018; O'Brien, K., 2012; Cave, E., 2011; Tingle, J., 2021; Glasper, A., 2021; Tingle, J., 2019; Byrom, A., 2013; Department of Health., 2002; National Patient Safety Agency., 2005; Department of Health., 2005; Department of Health., 2001; Francis, R., 2013; Berwick, D., 2013; General Medical Council., 2013; Dalton, D. and Williams, N., 2014; General Medical Council and Nursing and Midwifery Council., 2015; Department of Health., 2015b; Department of Health., 2015c; Francis, R., 2015b; NHS England and NHS Improvement., 2015; Ockenden,D., 2022; Kirkup, B., 2020; Royal College of Midwifery., 2022; World Health Organisation., 2005; Department of Health., 2007; National Patient Safety Agency., 2009; Parliamentary and Health Services Ombudsman., 2012; McHugh, S. et. al., 2024; NHS England., 2024;

Table 2: Records relating to how law and policy supports providers of NHS care in England to respond to patient harm grouped into initial codes and the contextual theme of professional behaviour.

Initial Code	Frequency	Records
Professional Duty of Candour	N=17	Vick, L., 2019; Francis, R., 2015a; Department of Health., 2015b; General Medical Council., 2013; Nursing and Midwifery Council., 2015; General Medical Council and Nursing and Midwifery Council., 2015; The Health and Care Professions Council., 2016; Lee v South Thames Regional Health Authority.,1985; Department of Health., 2000; Sokol, D., 2015; Vick, L., 2017; O'Brien, K., 2012; Francis, R., 2013; Francis, R., 2015b; NHS England and NHS Improvement., 2015; Kirkup, B., 2015; Ashurst, A., 2024;
Compassion	N=16	Glasper, A., 2021; MacDonald, M. et. al., 2014; Department of Health., 2009; NHS Employers., 2014; Department of Health., 2015c; Francis, R., 2015b; NHS England and NHS Improvement., 2019; National Patient Safety Agency., 2005; National Patient Safety Agency., 2009; Department of Health., 2009; Francis, R., 2013; General Medical Council., 2013; General Medical Council and Nursing and Midwifery Council., 2015; Ockenden,D., 2022; O’Dwyer, L., 2024; Robinson, A. et. al., 2025;
Professionalism	N=15	Thomas, E. and Leigh, B., 2014; O'Brien, K., 2012; Price-Davey, A.. 2017; Tingle, J., 2015a; Francis, R., 2013; General Medical Council., 2013; Dalton, D. and Williams, N., 2014; Department of Health., 2015b; Francis, R., 2015b; NHS England and NHS Improvement., 2015; Department of Health., 2009; Parliamentary and Health Services Ombudsman., 2012; General Medical Council and Nursing and Midwifery Council., 2015; Department of Health., 2015c; Kirkup, B., 2015;

Table 2: Records relating to how law and policy supports providers of NHS care in England to respond to patient harm grouped into initial codes and the contextual theme of professional behaviour.

Initial Code	Frequency	Records
Communication	N=13	Cave, E., 2011; O'Brien, K., 2012; Tingle, J., 2019; Parliamentary and Health Services Ombudsman., 2012; General Medical Council., 2013; Parliamentary and Health Services Ombudsman., 2015; Kirkup, B., 2015; Health and Social Care Act 2008 (Regulated Activities) Regulations 2014: Regulation 20; National Patient Safety Agency., 2005. National Patient Safety Agency., 2009; General Medical Council and Nursing and Midwifery Council., 2015; de Silva, P., 2025; Adams, M., 2024;
Supporting staff	N=11	Cruikshanks, A. and Bryden, C., 2018; Harrison, R. et. al., 2017; MacDonald, M. et. al., 2014; Parker, D. and Lawton, R., 2003; Thurtle, V., 2000; NHS Employers., 2014; Parliamentary and Health Services Ombudsman., 2015; Department of Health., 2015a; NHS England and NHS Improvement., 2019; Royal College of Midwifery., 2022; Edozien, L and Clements, R., 2006; Robinson, A. et. al., 2025;
Doctor-patient relationship	N=6	Cave, E., 2011; O'Brien, K., 2012; Basu, S. et. al., 2020; Tingle, J., 2019; Montgomery v Lanarkshire Health Board., 2015; Thomas, E. and Leigh, B., 2014;
Advocacy	N=6	Glasper, A., 2021; Tingle, J., 2016; General Medical Council and Nursing and Midwifery Council., 2015; Department of Health., 2015c; Ockenden,D., 2022; Department of Health., 2007;

Table 3: Records relating to how law and policy supports providers of NHS care in England to respond to patient harm grouped into initial codes and the contextual theme of investigation.

Initial Code	Frequency	Records
Learning from harm	N=58	Chaffer, D., 2021; Basu, S. et. al., 2020; Tingle, J., 2019; Cruikshanks, A. and Bryden, C. 2018; Price-Davey, A.. 2017; Harrison, R. et. al. , 2017; Eshareturi, C. and Serrant, L., 2017; Tingle, J., 2016; Tingle, J., 2015; Vincent, C. et. al., 2014; Macrae, C., 2014; MacDonald, M. et. al., 2014; Pinto, V. et. al., 2012; Tingle, J., 2010; Pearson, P. et. al., 2010; Wakefield, A. et. al., 2009; Benn, J.et. al., 2009; Astill, D., 2008; Lugon, M., 2000; Johnson, H. and Spencer, M., 2021; Vick, L., 2019; Vosper, H., 2018; Samuels, A., 2017; Blundell, V., 2015; O'Brien, K., 2012; Cave, E., 2011; Kennedy, I., 2001; Donaldson., L. 2003; Department of Health., 2003; National Patient Safety Agency., 2004; Department of Health., 2001; World Health Organisation., 2005; Department of Health., 2006; National Patient Safety Agency., 2009; Department of Health., 2007; National Patient Safety Agency., 2005; Department of Health., 2009; Department of Health., 2000; Parliamentary and Health Services Ombudsman., 2012; Parliamentary and Health Services Ombudsman., 2015; Department of Health., 2015a; NHS Resolution., 2018; Department of Health., 2015b; Department of Health., 2015c; Francis, R., 2015b; NHS England and NHS Improvement., 2015; Kirkup, B., 2015; NHS Resolution., 2019; NHS Resolution., 2022; NHS England and NHS Improvement., 2019; Kirkup, B., 2020; Getting It Right First Time and NHS Resolution., 2021; National Audit Office., 2005; ; de Silva, P., 2025; McHugh, S. et. al., 2024; Adams, M., 2024; Robinson, A. et. al., 2025; NHS England., 2024;

Table 3: Records relating to how law and policy supports providers of NHS care in England to respond to patient harm grouped into initial codes and the contextual theme of investigation.

Initial Code	Frequency	Records
Complaint response	N=22	Blundell, V., 2015; Johnson, H. and Spencer, M., 2021; Cave, E., 2011; Burn, S., 2003; Passmore, K., 2002; National Audit Office., 2005; National Patient Safety Agency., 2005; Kirkup, B., 2020; Francis, R., 2013; Parliamentary and Health Services Ombudsman., 2015; Department of Health., 2015c; Francis, R., 2015b; Cruikshanks, A. and Bryden, C., 2018; Department of Health., 2001; Department of Health., 2007; National Patient Safety Agency., 2009; Department of Health., 2009; Parliamentary and Health Services Ombudsman., 2012; Nursing and Midwifery Council., 2015; Department of Health., 2015b; Kirkup, B., 2015; NHS England., 2024;
Incident response	N=24	Cruikshanks, A. and Bryden, C., 2018; Eshareturi, C. and Serrant, L., 2017; MacDonald, M. et. al., 2014; Astill, D., 2008; Donaldson., L. 2003; National Patient Safety Agency., 2004; National Audit Office., 2005; Johnson, H. and Spencer, M., 2021; Parliamentary and Health Services Ombudsman., 2015; Department of Health., 2015a; NHS England and NHS Improvement., 2015; NHS England and NHS Improvement., 2019; Department of Health., 2003; Department of Health., 2001; General Medical Council and Nursing and Midwifery Council., 2015; Royal College of Midwifery., 2022; Department of Health., 2000; Nichols, E., 2024; de Silva, P., 2025; McHugh, S. et. al., 2024; O’Dwyer, L., 2024; Kirwan, H. and McNeil, P., 2024; NHS England., 2024; NHS England., 2022;
Public Inquiries	N=15	Cowan, J., 2003; Passmore, K., 2002; Henderson, N., 2001a; Henderson, N., 2001b; National Audit Office., 2005; Department of Health., 2007; Inquiries Act., 2005; Department of Health., 2015b; Kirkup, B., 2015; Ockenden,D., 2022; Kirkup, B., 2020; Johnson, A., 2021; Tingle, J., 2019; Department of Health., 2001; Department of Health., 2000; de Silva, P., 2025;

Table 4: Records relating to how law and policy supports providers of NHS care in England to respond to patient harm grouped into initial codes and the contextual theme of resolution.

Initial Code	Frequency	Records
Learning from harm	N=32	Cruikshanks, A. and Bryden, C., 2018; Price-Davey, A.. 2017; Harrison, R. et. al. , 2017; Draycott, T. et. al., 2015; Tingle, J., 2015b; MacDonald, M. et. al., 2014; Pearson, P. et. al., 2010; Wakefield, A. et. al., 2009; Benn, J.et. al., 2009; Nursing and Midwifery Council., 2015; Vosper, H., 2018; Samuels, A., 2017; Cave, E., 2011; Donaldson., L. 2003; National Patient Safety Agency., 2004; Department of Health., 2006; Department of Health., 2011; Department of Health., 2007; Parliamentary and Health Services Ombudsman., 2012; Berwick, D., 2013; Dalton, D. and Williams, N., 2014; Parliamentary and Health Services Ombudsman., 2015; Department of Health., 2015b; Department of Health., 2015c; NHS England and NHS Improvement., 2015; Kirkup, B., 2015; NHS Resolution., 2022; NHS England and NHS Improvement., 2019; Kirkup, B., 2020; Getting It Right First Time and NHS Resolution., 2021; Department of Health., 2000; Nichols, E., 2024; Ashurst, A., 2024;
Apology	N=22	Awais, Z., 2015; O'Brien, K., 2012; Cruikshanks, A. and Bryden, C., 2018; National Patient Safety Agency., 2004; Department of Health., 2006; General Medical Council., 2013; NHS England and NHS Improvement., 2015; General Medical Council and Nursing and Midwifery Council., 2015; NHS Resolution., 2019; NHS Resolution., 2022; National Audit Office., 2001; National Patient Safety Agency., 2009; Glasper, A., 2021; Chaffer, D., 2021; Department of Health., 2003; Department of Health., 2009; Department of Health., 2015c; Kirkup, B., 2015; Parliamentary and Health Services Ombudsman., 2016; de Silva, P., 2025; Ashurst, A., 2024; Adams, M., 2024;
Alternative dispute resolution	N=12	Vernon, H., 2019; Awais, Z., 2015; Thomas, E. and Leigh, B., 2014; Cave, E., 2011; Burn, S., 2003; NHS Litigation Authority., 2001; Dimond, B., 2001; Walker, D. and Punt, J., 2022; National Audit Office., 2001; Donaldson., L. 2003; NHS Redress Act., 2006; Department of Health., 2001;

Table 4: Records relating to how law and policy supports providers of NHS care in England to respond to patient harm grouped into initial codes and the contextual theme of resolution.

Initial Code	Frequency	Records
Complaint resolution	N=7	Cave, E., 2011; Cruikshanks, A. and Bryden, C., 2018; Tingle, J., 2016; Tingle, J., 2010; National Patient Safety Agency., 2005; National Patient Safety Agency., 2009; O’Dwyer, L., 2024;

Table 5: Records relating to how law and policy supports providers of NHS care in England to respond to patient harm grouped into initial codes and the contextual theme of organisational culture.

Initial Code	Frequency	Records
Culture of candour	N=46	Edozien, L and Clements, R., 2006; Vosper, H., 2018; O'Brien, K., 2012; Tingle, J., 2015a; Tingle, J., 2015b; Kirkup, B., 2015; Pinto, V. et. al., 2012; Tingle, J., 2010; Kennedy, I., 2001; Department of Health., 2002; Department of Health., 2001; National Audit Office., 2005; Francis, R., 2013; Berwick, D., 2013; General Medical Council., 2013; Dalton, D. and Williams, N., 2014; NHS Employers., 2014; Parliamentary and Health Services Ombudsman., 2015; Department of Health., 2015a; Nursing and Midwifery Council., 2015; General Medical Council and Nursing and Midwifery Council., 2015; Department of Health., 2015b; Department of Health., 2015c; Francis, R., 2015b; NHS England and NHS Improvement., 2015; Health and Social Care Act 2008 (Regulated Activities) Regulations 2014: Regulation 20; Parliamentary and Health Services Ombudsman., 2016; The Health and Care Professions Council., 2016; Beatt v Croydon Health Services NHS Trust., 2017; NHS Resolution., 2019; NHS England and NHS Improvement., 2019; Kirkup, B., 2020; Getting It Right First Time and NHS Resolution., 2021; Royal College of Midwifery., 2022; National Patient Safety Agency., 2004; Department of Health., 2003; National Patient Safety Agency., 2005. National Patient Safety Agency., 2009; Kirkup, B., 2015; NHS Resolution., 2018; de Silva, P., 2025; McHugh, S. et. al., 2024; Adams, M., 2024; O’Dwyer, L., 2024; Kirwan, H. and McNeil, P., 2024;

Table 5: Records relating to how law and policy supports providers of NHS care in England to respond to patient harm grouped into initial codes and the contextual theme of organisational culture.

Initial Code	Frequency	Records
Patient involvement	N=33	Tingle, J., 2016; Vincent, C and Coulter, A.,2002; Department of Health., 2003; Donaldson., L. 2003; National Audit Office., 2005; Department of Health., 2006; National Patient Safety Agency., 2005; Department of Health., 2009; Parliamentary and Health Services Ombudsman., 2012; Francis, R., 2013; Berwick, D., 2013; General Medical Council., 2013; Montgomery v Lanarkshire Health Board., 2015; Parliamentary and Health Services Ombudsman., 2015; Department of Health., 2015a; Nursing and Midwifery Council., 2015; Department of Health., 2015c; NHS England and NHS Improvement., 2015; NHS Resolution., 2019; NHS Resolution., 2018; NHS Resolution., 2022; NHS England and NHS Improvement., 2019; Kirkup, B., 2020; Getting It Right First Time and NHS Resolution., 2021; Price-Davey, A.. 2017; Department of Health., 2007; National Patient Safety Agency., 2009; Department of Health., 2015b; NHS Resolution., 2019; de Silva, P., 2025; McHugh, S. et. al., 2024; Adams, M., 2024; NHS England., 2024;
Freedom to Speak Up / Whistleblowing	N=23	Vick, L., 2017; Blundell, V., 2015; Awais, Z., 2015; Glasper, A., 2021; Tingle, J., 2015; Kirkup, B., 2015; Macrae, C., 2014; MacDonald, M. et. al., 2014; Byrom, A., 2013; Tasker, A., Jones, J. and Brake, S., 2023; Department of Health., 2002; Department of Health., 2001; Department of Health., 2011; Montgomery v Lanarkshire Health Board., 2015; Department of Health., 2015a; Department of Health., 2015b; Francis, R., 2015; NHS England and NHS Improvement., 2015; Parliamentary and Health Services Ombudsman., 2016; The Health and Care Professions Council., 2016; Getting It Right First Time and NHS Resolution., 2021; Royal College of Midwifery., 2022; NHS England and NHS Improvement., 2019; de Silva, P., 2025;

Table 5: Records relating to how law and policy supports providers of NHS care in England to respond to patient harm grouped into initial codes and the contextual theme of organisational culture.

Initial Code	Frequency	Records
Safety culture	N=22	Cowan, J., 2003; Parker, D. and Lawton, R., 2003; Passmore, K., 2002; Lugon, M., 2000; Thurtle, V., 2000; Tasker, A., Jones, J. and Brake, S., 2023; General Medical Council., 2013; General Medical Council and Nursing and Midwifery Council., 2015; Department of Health., 2015b; Department of Health., 2015c; NHS Resolution., 2018; NHS Resolution., 2019; Vosper, H., 2018; Pearson, P. et. al., 2010; Benn, J.et. al., 2009; Department of Health., 2000; Johnson, H. and Spencer, M., 2021; National Patient Safety Agency., 2004; Berwick, D., 2013; General Medical Council and Nursing and Midwifery Council., 2015; NHS England., 2024;
Blame culture	N=22	Edozien, L and Clements, R., 2006; Parker, D. and Lawton, R., 2003; Passmore, K., 2002; Lugon, M., 2000; Tasker, A., Jones, J. and Brake, S., 2023; National Patient Safety Agency., 2004; National Patient Safety Agency., 2005. Department of Health., 2001; Department of Health., 2006; Getting It Right First Time and NHS Resolution., 2021; Royal College of Midwifery., 2022; Department of Health., 2003; World Health Organisation., 2005; National Patient Safety Agency., 2009; Berwick, D., 2013; Getting It Right First Time and NHS Resolution., 2021; Department of Health., 2000; Nichols, E., 2024; de Silva, P., 2025; McHugh, S. et. al., 2024; Devlin, M., 2024; Kirwan, H. and McNeil, P., 2024;

Table 5: Records relating to how law and policy supports providers of NHS care in England to respond to patient harm grouped into initial codes and the contextual theme of organisational culture.

Initial Code	Frequency	Records
Staff support	N=18	Cruikshanks, A. and Bryden, C., 2018; Harrison, R. et. al. , 2017; MacDonald, M. et. al., 2014; Parker, D. and Lawton, R., 2003; Thurtle, V., 2000; Department of Health., 2009; NHS Employers., 2014; Parliamentary and Health Services Ombudsman., 2015; Department of Health., 2015b; NHS England and NHS Improvement., 2019; Royal College of Midwifery., 2022; Edozien, L and Clements, R., 2006; Department of Health., 2006; Lugon, M., 2000; McHugh, S. et. al., 2024; Adams, M., 2024; Robinson, A. et. al., 2025; NHS England., 2024;
Learning culture	N=14	Cruikshanks, A. and Bryden, C., 2018; Harrison, R. et. al. , 2017; MacDonald, M. et. al., 2014; Parker, D. and Lawton, R., 2003; Thurtle, V., 2000; General Medical Council., 2013; Parliamentary and Health Services Ombudsman., 2015; NHS England and NHS Improvement., 2015; NHS England and NHS Improvement., 2019; Royal College of Midwifery., 2022; Edozien, L and Clements, R., 2006; Department of Health., 2006; National Patient Safety Agency., 2005; National Patient Safety Agency., 2009;
Incentivisation	N=14	Draycott, T. et. al., 2015; Sokol, D., 2015; Tingle, J., 2015; Vincent, C. et. al., 2014; Parliamentary and Health Services Ombudsman., 2012; Berwick, D., 2013; NHS Employers., 2014; Nursing and Midwifery Council., 2015; Francis, R., 2015; NHS Resolution., 2018; NHS England and NHS Improvement., 2019; Department of Health., 2011; Awais-Bilal, Z., 2015; Adams, M., 2024;
Culture of fear	N=14	Awais-Bilal, Z., 2015; Samuels, A., 2017; Vick, L., 2017; Cave, E., 2011;Thomas, E. and Leigh, B., 2014; Tingle, J., 2019; Pearson, P. et. al., 2010; Henderson, N., 2001b; Department of Health., 2001; NHS Resolution., 2019; NHS England and NHS Improvement., 2019; Kirkup, B., 2020; McHugh, S. et. al., 2024; O'Dwyer, L., 2024;

Table 5: Records relating to how law and policy supports providers of NHS care in England to respond to patient harm grouped into initial codes and the contextual theme of organisational culture.

Initial Code	Frequency	Records
No-blame culture	N=13	Edozien, L and Clements, R., 2006; Price-Davey, A.. 2017; MacDonald, M. et. al., 2014; Pearson, P. et. al., 2010; Astill, D., 2008; Parker, D. and Lawton, R., 2003; Passmore, K., 2002; Lugon, M., 2000; Tasker, A., Jones, J. and Brake, S., 2023;National Patient Safety Agency., 2005; Department of Health., 2015; Kirkup, B., 2015; Francis, R., 2015;
Just culture	N=13	Vosper, H., 2018; O'Brien, K., 2012; Glasper, A., 2021; Benn, J.et. al., 2009; Tasker, A., Jones, J. and Brake, S., 2023; National Audit Office., 2005; Francis, R., 2015; NHS England and NHS Improvement., 2015; NHS Resolution., 2019; NHS England and NHS Improvement., 2019; Royal College of Midwifery., 2022; McHugh, S. et. al., 2024; Robinson, A. et. al., 2025;
Culture of defensiveness	N=11	Cave, E., 2011; Tingle, J., 2019; Dimond, B., 2001; Case, P., 2020; Vosper, H., 2018; Passmore, K., 2002; Whitehouse v Jordan., 1981; Department of Health., 2001; Parliamentary and Health Services Ombudsman., 2015; Department of Health., 2015a; Department of Health., 2015b;
Culture of denial	N=10	O'Brien, K., 2012; Cave, E., 2011; Vick, L., 2019; Blundell, V., 2015; Tingle, J., 2019; Kirkup, B., 2015; Parker, D. and Lawton, R., 2003; NHS England and NHS Improvement., 2015; Department of Health., 2015b; Francis, R., 2015;
Reporting culture	N=10	Benn, J.et. al., 2009; Tasker, A., Jones, J. and Brake, S., 2023; Kennedy, I., 2001; National Patient Safety Agency., 2005; Department of Health., 2006; National Audit Office., 2005; Department of Health., 2015a; Department of Health., 2015b; NHS Resolution., 2019; Royal College of Midwifery., 2022;

Table 5: Records relating to how law and policy supports providers of NHS care in England to respond to patient harm grouped into initial codes and the contextual theme of organisational culture.

Bullying culture	N=7	Vick, L., 2019; Tingle, J., 2019; Sokol, D., 2015; Tasker, A., Jones, J. and Brake, S., 2023; Department of Health., 2015b; Kirkup, B., 2015; NHS Resolution., 2019;
Fair culture	N=7	Awais, Z., 2015; Parker, D. and Lawton, R., 2003; Tasker, A., Jones, J. and Brake, S., 2023; Department of Health., 2006; National Audit Office., 2005; National Patient Safety Agency., 2009;NHS England and NHS Improvement., 2015;
Compensation culture	N=5	Cave, E., 2011; O'Brien, K., 2012; Dimond, B., 2001; Burn, S., 2003; Department of Health., 2011;
Professional culture	N=5	O'Brien, K., 2012; Macrae, C., 2014; Wakefield, A. et. al., 2009; Department of Health., 2001; Kennedy, I., 2001;
